# Upstreaming and Normalizing Advance Care Planning Conversations—A Public Health Approach

**DOI:** 10.3390/bs7020018

**Published:** 2017-04-12

**Authors:** Maryjo Prince-Paul, Evelina DiFranco

**Affiliations:** School of Nursing, Case Western Reserve University, 2120 Cornell Road, Cleveland, OH 44106, USA; evelina.difranco@case.edu

**Keywords:** advance care planning, death education, public health

## Abstract

As a society, we simply don’t talk about this universal experience called dying and death; in fact, we ignore it until we have to face it. Thus, it is often in a crisis experience when we have to make decisions while we are laden with uncertainty and intense emotions. Sixty percent of people say making sure their family is not burdened by tough decisions is extremely important, yet 56% of them have not held a conversation about its context. Instead of waiting to make end-of-life decisions, let us begin to think about what matters most while we are living, what we value most, and how we translate these values into conversations about what is important. As a public health concern, if we can upstream the advance care planning discussion into usual health promotion activities, perhaps, as a society, we can begin to normalize and reshape how we make decisions about the last chapters of our lives.

## 1. Introduction

The inevitability of death encompasses us all. We are all born with the disease of mortality. We all die. And yet, to many of us, the details of dying and death are a mystery. It is an abstraction we would rather not think about. Contemplating our own death and doing the necessary preparatory work is a rarity in modern America. In fact, most all people in modern America think the time to decide about health care choices is “later.” Furthermore, having end-of-life discussions are rarely considered during the routine days of living; rather, these types of conversations and health care decisions are made during a time of crisis, when both patients and families are laden with anxiety, fear, and the lack of time to make mindful, reasonable, and logical decisions. This lack of openness has affected the quality and range of support and care services available to patients and their families. It has also affected our ability to die where or how we would wish. As Americans, we should learn how to make a place for death in our lives and learn how to plan for it.

A 2013 national survey of nearly 2100 Americans aged 18 and older found that, while 90% said that talking about end-of-life decisions was important, fewer than 30% had actually done so [1]. In another survey, 82% of people said it was important to put their wishes in writing, yet only 23% had actually done it [2,3]. The fact is that when patients with serious and life-threatening illness are prepared, they die well and their families and caregivers tend to grieve better. When people have done the work of considering their own goals and values and have documented those preferences, they make different choices.

Modern medicine in America saves many people from acute illness, who then go on to live longer with chronic illnesses whose disease trajectories are associated with declining physical and mental functioning over months and years, often punctuated by episodes of acute illness and decompensation. Unfortunately, unnecessary suffering, as well as dissatisfaction with and poor health care resource utilization result from a mismatch between the patients and family’s needs and the current health care system environment. In most cases, the suffering could be avoided, or at least mitigated, by some education on dying and death and informed conversations about it. Ultimately, this will involve a fundamental change in society in which dying, death, and bereavement will be thought about, seen, and accepted as a natural part of life’s cycle. Upstreaming Advance Care Planning (ACP) and its accompanying discussions provides a means of ameliorating this mismatch, but is yet to be embedded in America’s public consciousness.

## 2. What is Advance Care Planning

Advance care planning (ACP) is a process of communication between individuals, families, and others who are considered important to the discussion, as well as health care providers, to understand, discuss, and plan future health care decisions, not only to lay preparations in the event that an individual loses decision-making capacity, but also to offer detailed instruction about values and wishes. ACP is about planning and talking about the “what ifs” that might occur across the entire lifespan. The goal is to try to engage in conversations more proactively rather than just reacting to changes in health conditions.

Advance directives are one part of the advance care planning process; that is, a formal, legal document that addresses plans about what treatment people wish to have or not have when they near death. These statements can include expectations about what people may wish to refuse to have, such as cardiopulmonary resuscitation, artificial ventilation, or artificial nutrition/feeding; they can be positive preferences such as what they would like to experience when they are near death, such as being at home with a loved one, preserving dignity and worth, and leaving a legacy. In addition, this process should involve the identification of a decision-maker, or surrogate decision-maker, who will honor, uphold, and respect a person’s preferences. However, this document goes into effect only when a person is incapacitated and loses the ability to speak for him/herself. Most importantly, this document should be viewed as a “living document”—one that can be revised and adjusted over time, as situations change, including change in health status.

## 3. Advance Care Planning as a Process

In the past, ACP has often been focused on the completion rates of actual advance directive documents, despite the lack of evidence to support that such documents improve end-of-life care or correspond with future care preferences [4]. Although evidence remains insufficient that ACP documentation leads to engagement of health care professionals in end-of-life discussions, we argue that upstreaming these conversations into lay communication may heighten the “normalization” of the topic into mainstream dialogue [5].

Perhaps a more superior focus will encourage widespread dialogue about ACP as a process for iteratively identifying and facilitating what people constitute as a “good death”, including identifying what factors are considered important (i.e., achieving a sense of control, leaving a legacy, maintaining a sense of dignity, being without pain or symptoms, relieving financial burdens, strengthening close relationships, and saying important things), and for informally communicating their future wishes [6]. Fried and colleagues suggest that ACP should be recognized as a health behavior and that the most effective way to engage people in this process is to tailor the information to a person’s readiness for engagement [7].

Conceptually, this comprises five distinct phases, from pre-contemplation to action and maintenance, which includes the completion of a written advance directive (a living will and a durable power of attorney for health care, otherwise known as a surrogate decision-maker). Three necessary components are germane to this discussion. First, there must be a willingness of the individual to reflect. This involves a discussion aimed at defining values, life goals, and wishes about the future. Commonly, this is grounded in how one sees a “life well lived”. Second, there is need for an organized “coming together” of all persons who will be involved in honoring the wishes. Plain language, timing, and trust are key elements of the success of this meeting. Third, an ongoing discussion about the preferences, especially in light of the complexity of life-limiting and serious illness, must be engaged in [8,9]. Conversations take time and effort and cannot be completed as a single checklist; they need to take place on more than one occasion.

As outlined in the standards of the National Framework and Preferred Practices for Palliative and Hospice Care Quality (NQF) optimal advance care planning is not a one-time event, but an ongoing discussion at critical milestones throughout the life cycle (e.g., when a person turns 18 years of age) [10,11]. Initiating these conversations earlier in the life cycle, at key maturation points, presumes that the person is generally healthy and has decision-making capacity. This can normalize discussion about values and life goals that can be revisited overtime, as part of primary health care, or simply when having conversation within the family context during sentinel life events. Ideally, these discussions would start early in adulthood, addressing global values and the selection of potential proxy decision-makers. With changes in health status, they would reflect more specific instructions.

Challenges of ACP derive from both a sociological and technological perspective. From a sociological context, there is a pervasive reluctance to publicly and personally engage in discussion about how people want to live with a serious illness, how they want to personally engage in discussion about dying and death, and how they would prefer to be cared for at the end of their lives. In addition, there are diverse ethnic and religious understandings, teachings, and preferences about individual autonomy. From a technological lens, different types of diseases have different disease trajectories and treatment options often have varying purposes with often contrasting consequences. And, while some older adults remain healthy and robust until very close to death, it is more likely that an older individual will have lived for two or more years with one or more chronic diseases and will have experienced substantial disability before dying. Along the way, he or she, and the family, will have to make what are sometimes difficult choices about health care.

## 4. Our Aging Population in the USA

Throughout our lives, but especially when we are older and facing increased risk of serious illness, we need a plan about what services are essential to living well and meaningfully. Medical advancements have contributed to increased life expectancy for Americans. The number and proportion of older persons in the United States is rapidly increasing. Persons 65 years or older numbered 46.2 million in 2014 (the latest year for which data is available). They represent 14.5% of the U.S. population, which is about one in every seven Americans. By 2060, there will be nearly 98 million older persons, more than twice their number in 2014. People 65+ represented 14.5% of the population in the year 2014, but are expected to grow to be 21.7% of the population by 2040 and the youngest members of the Baby Boomer generation will reach 65 years of age in 2030 [12]. Taken together, the unprecedented numbers of aging adults coupled with the corresponding likelihood of chronic conditions, such as heart disease, diabetes, dementia, depression, frailty, and end-of-life issues, will challenge the existing health care system. As the face of America ages, holding conversations about preferences for care is therefore paramount. Most often with family present, elders do engage in ACP conversation, if given the opportunity to reflect and share. Those who have had this conversation are almost three times as likely to have their end-of-life wishes both known and followed, and their family members demonstrate less anxiety, stress, and depression during bereavement [13].

## 5. ACP and Public Health, Education, Engagement

Internationally, death awareness and death literacy are not only more culturally transparent, but seem to be integrated into the context of everyday living. Through community engagement and social action, conversations about death and dying are commonplace and have set the stage for the development of a public health approach, specifically in such countries as the United Kingdom and Australia [5]. Death literacy, defined as a set of knowledge or skills that help persons gain access to, understand, and then act upon end-of-life and death care options, is positioned within a public health framework [14]. It is a resource that people and communities use to strengthen their capacity for future caring. Embedded in this framework is death education, and its role is to moderate the relationship between death awareness and knowledge about society as a death system. Taken together, this public heath approach, commonly practiced in the aforementioned countries, is operationalized through community engagement, collaboration, and empowerment, and creates a template for an American public health approach to ACP.

The Centers for Disease Control and Prevention (CDC) recognizes the public health opportunity to educate Americans, especially older adults, about ACP in order to improve their quality of care at the end of life [15]. ACP also meets other criteria that define a public health issue. According to the CDC, ACP can potentially affect a large number of people, can reduce unwanted, futile, and expensive treatment, and can meet public demand to change the way care has been addressed in the past. Just as health care is not solely the responsibility of the sick but also the healthy, so too, dying and death are the responsibility of everyone, not simply those who are old or have serious illness. In order to provide a context for the role of public health engagement, it is critical to first establish what must happen before this movement gains momentum.

A public health education approach to death and dying can upstream the conversation about ACP squarely in the domain of a broader death education context. Not disseminating general education about death and dying (having open discourse about this inevitability) and/or encouraging conversations about ACP, and then leaving a loved one to make critical decisions for their sick family member, is like asking people to eat healthier (planning meals, recognizing healthier options) without providing education on the nutritional value in the food products they are purchasing or resources on planning meals and better habits around eating. By engaging schools, workplaces, service clubs, recreation facilities, churches and their leaders, and other venues (see Box 1), death education becomes a population health approach for health promotion. This action has the momentum to not only change social attitudes, but also the behaviors and qualities of experiences of living until death.

Box 1 **Venues to consider for public education and conversation about advance care planning.**

- Churches, synagogues, temples, and other places of worship (and their leaders)
- Service Clubs (Rotary International, Kiwanis, Lions)
- Local public library forums
- Girls Scouts and Boys Scouts of America meetings (Merit badges—e.g., public health, family life, communication, law)
- Book Clubs
- Senior Centers
- Local fitness centers
- Barber shops/beauty salons
- High school curricula (http://www.dyingmatters.org)
- Undergraduate courses at public and private schools of higher education
- Death cafes (http://www.deathcafe.com)
- Wellness programs at places of employment
- Progressive dinners/Death Over Dinner (http://deathoverdinner.org)
- National Healthcare Decisions Day (http://www.nhdd.org)

Recommendation 5 of the Institute of Medicine’s report on Improving Quality and Honoring Individual Preferences Near the End of Life highlights the importance of public education and engagement [16]. It states: “Civic leaders, public health and other governmental agencies, community-based organizations, faith-based organizations, consumer groups, and professional societies, should engage their constituents and provide fact based information to encourage ACP and informed choice based on the needs and values of individuals. Public education and engagement efforts should aim to normalize these difficult conversations and to assist people in achieving the necessary information to have meaningful discussions about the values and goals of care” [16] (p. 370). Like all modern public health initiatives, the pursuit of death education and engagement programs in the community should seek to create social changes that promote healthy behaviors, reduce harm, and maximize well-being and quality of life.

## 6. One Avenue: The Influence of Community Clergy

As the baby boomer generation ages, increased numbers of persons will inevitably be forced to cope with illness and end-of-life issues, bringing diverse cultural and spiritual beliefs and practices into making decisions about how they want to live until they die. Religious and/or spiritual beliefs remain central to most Americans; they provide a sense of continuity of self and a sense of belonging, especially in the face of serious illness [17]. Today, the role of religion and spirituality has become an increasingly salient component as people aim to find a sense of connectedness and purpose before life’s end [18]. America’s current population not only reflects an aging population but one with multigenerational family members combined with an array of spiritual practices. Unfortunately, spiritual practice and its integration in the health care delivery system is often overlooked [19]. Evidence suggests that many people want spirituality incorporated as a component of health care, but most report that spiritual needs are often neglected by the medical community [20]. A sense of meaning and purpose in life, supported by spirituality is related to lower death anxiety, death avoidance, and depression, and an overall sense of greater subjective well-being [21]. Community clergy, spiritual leaders, and places of worship have a unique opportunity to engage constituents, including families, into conversations about ACP before illness strikes [22]. Spiritual leaders are situated in a relationship of trust with covenants and they have an important role to help clarify ways in which people’s beliefs and values might influence their health care preferences and decisions.

## 7. National Movements at the Community Level

Unfortunately, we live in a society that largely denies death or at least attempts to avoid it. Yet, it is the case that most Americans will age and die; there is a finitude of living. The reluctance to examine this experience shapes the way we view and think about dying well. However, many Americans tell stories about death gone wrong and how their parents or other family members received care that was inconsistent with their values and wishes. This has activated consumers and generated an approach about not accepting care that violates their own wishes. By sharing these very personal stories in the public domain, people have started a national conversation that is creating a dynamic, social shift.

Community engagement programs have the capacity to mobilize and maximize family, community, and workplace supports in an effort to reorganize a culture of denial toward a culture of acknowledgement of this universal experience. The current national conversation to encourage the general public to talk more about death and dying or, more specifically, what is valued the most, should greatly facilitate ACP. Two recent, national efforts have largely propelled the dialogue—The Conversation Project and the Stanford Letter Project [1,23].

The Conversation Project is a public engagement campaign that advocates “kitchen table” conversations with family and friends about wishes and preferences for health care [1]. The Conversation Project, in collaboration with the Institute to Improve Health Care (IHI), offers people the tools and guidance by way of the Conversation Starter Kit—a resource organized by a “get ready, get set, go, keep going” approach that reflects the Transtheoretical Model outlined by Fried and colleagues [7]. Intended to specifically gather individuals’ preferences for end-of-life care, the Conversation Project’s campaign may be casting a larger net, from a public health perspective. Social support has been shown to have the greatest influence on health- related quality of life outcomes [24]. By gathering loved ones, friends, and people who matter most around a kitchen table or a common meeting area, social engagement and support occur organically. Perhaps these difficult discussions will become easier and more comfortable when taking place with important others, before a crisis, and in the comfort of a natural surrounding—not the intensive care unit.

Another effort, the Stanford Letter project, began in 2015 under the direction of Dr. Vyjeyanthi Periyakoil from Stanford University School of Medicine [23]. Dr. Periyakoil and her team spent years conducting interviews and focus groups in multiple languages with people in the community and talking to numerous patients and their families about the challenges of having and preparing for discussions about the last phase of life. Their research has shown that most Americans find it extremely difficult to discuss this important topic with both their family members and friends, as well as their health care providers. Furthermore, people simply do not quite know how to initiate these conversations [25]. To that end, the Letter Project and its accompanying tools were specifically designed to help people voice key information needed to prepare for the future. Three letter templates exist and include: “The What Matters Most” template—a document that provides anyone the space to write about what matters most to them and what treatments they want in the future; the “Letter Project Advance Directive”—a valid advance directive and a supplemental letter that describes preferences for medical care at the end of life and is submitted to the health care provider; and the “Friends and Family Letter”—a life review document that acknowledges important people, treasured moments, and allows for sharing relational-based conversation including gratitude, love, and forgiveness. The Stanford Letter Project goal is to help, empower, and support all adults to prepare for their future and to take the initiative to talk to their doctors and their friends and family about what matters most to them at life’s end. All tools are free and available in print, as an online fillable form, and as a mobile app.

## 8. Conclusions

For most people in the United States, until a loved one is actually facing a serious, life-threatening illness, interest in engaging in ACP discussions is often low. The demands of everyday living coupled with our pervasive societal denial of death in the United States, provide a ready excuse to not engage. Upstreaming ACP conversations will require a broad participation of multiple stakeholders, not limited to health care providers. We must stretch to the public health, social and supporting services sector, such as faith based communities where Americans and their families often rely on assistance for practical issues, information, and advice. As a result, we can potentially transform our culture so that more people can have their values and preferences about what matters most to them honored at life’s end. Perhaps then, as a society, we will have the courage to confront the reality of mortality and to seek the truth about our hopes and our fears.
